# The Effect of a New Coating on the Drying Performance of Fruit and Vegetables Products: Experimental Investigation and Artificial Neural Network Modeling

**DOI:** 10.3390/foods9030308

**Published:** 2020-03-09

**Authors:** S. M. Atiqure Rahman, Ahmed M. Nassef, Mujahed Al-Dhaifallah, Mohammad Ali Abdelkareem, Hegazy Rezk

**Affiliations:** 1 Dept. of Sustainable and Renewable Energy Engineering, University of Sharjah, Sharjah 27272, UAE; mabdulkareem@sharjah.ac.ae; 2 College of Engineering at Wadi Addawaser, Prince Sattam Bin Abdulaziz University, Al-Kharj 16278, KSA; a.nasef@psau.edu.sa (A.M.N.); hegazy.hussien@mu.edu.eg (H.R.); 3 Computers and Automatic Control Engineering Department, Faculty of Engineering, Tanta University, Tanta 31512, Egypt; 4 Systems Engineering Department, King Fahd University of Petroleum & Minerals, Dhahran 31261, KSA; 5 Center of Advanced Materials Research, Research Institute of Science and Engineering, University of Sharjah, Sharjah 27272, UAE; 6 Chemical Engineering Department, Minia University, Minya 61519, Egypt; 7 Electrical Engineering Department, Faculty of Engineering, Minia University, Minya 61519, Egypt

**Keywords:** drying performance, alginic and polygalacturonic acid, glucose and sucrose, edible coating, osmotic dehydration, artificial neural network, modeling

## Abstract

A study on mass transfer using new coating materials (namely alginic acid and polygalacturonic acid) during osmotic dehydration—and hence in a laboratory-scale convective dryer to evaluate drying performance—was carried out. Potato and apple samples were examined as model heat-sensitive products in this study. Results indicate that the coating material containing both alginic acid and polygalacturonic acid causes higher water loss of about 17% and 7.5% and lower solid gain of about 4% and 8%, respectively, compared to uncoated potato sample after a typical 90 min osmotic dehydration process. Investigation of drying performance using both coating materials showed a higher reduction in the moisture content of about 22% and 18%, respectively, compared with uncoated samples after the 3 h drying period. Comparisons between the two proposed coating materials were also carried out. Samples (potato) coated with alginic acid demonstrated better performance in terms of higher water loss (WL), lower solid gain (SG), and notable enhancement of drying performance of about 7.5%, 8%, and 8%, respectively, compared to polygalacturonic acid. Similar outcomes were observed using apple samples. Additionally, an accurate model of the drying process based on the experimental dataset was created using an artificial neural network (ANN). The obtained mean square errors (MSEs) for the predicted water loss and solid gain outputs of the potato model were 4.0948e^−5^ and 3.924e^−6^, respectively. However, these values for the same parameters were 3.164e^−5^ and 4.4915e^−6^ for the apple model. The coefficient of determination (*r*^2^) values for the two outputs of the potato model were found to be 0.99969 and 0.99895, respectively, while they were 0.99982 and 0.99913 for the apple model, which reinforces the modeling phase.

## 1. Introduction

The continuous increase in food demand throughout the year has led to food products being preserved for a longer time. The surrounding temperature and internal moisture content often affect the longevity of food products [1]. Osmotic dehydration is a complex dynamic mass transfer process that has been widely used as a pretreatment procedure in the food drying process to maintain or improve food quality [2,3,4,5,6,7]. This is the process where water is partially removed through immersion of the solid-containing-water in an aqueous solution. It is a useful drying technique that is used prior to a regular drying process. Sugar and salt aqueous solutions at high osmotic pressure are some of the suitable options for osmotic drying of fruits and vegetables, whereby the solid product either (whole or in pieces) is placed into the solution [8,9]. This gives rise to at least two major simultaneous counter-current flows, namely water flowing out of the food into the solution and a simultaneous transfer of a solute from the solution into the food, which are both due to water and solute activity gradients across the cell membrane [3,10,11,12]. 

Regardless of the benefits of osmotic dehydration, a solid film beneath the surface of the product is formed as a result of solid uptake due to the concentrated solid solution. Hence, it reduces the osmotic pressure gradient through the product-medium interface and decreases the dynamics force for water flow from the product to the osmotic solution. In addition, the solid uptake clogs the path of moisture diffusion from the inner part of the product to the outer surface, and thus decreases the dehydration rate during the subsequent osmotic process, as well as in the drying process.

To overcome this problem, the edible coating could be applied before the osmotic process takes place. Edible coatings are thin layers of edible materials applied to the product surface that efficiently obstruct the penetration of the solute inside the food. Therefore, more efficient osmotic dehydration could be obtained by coating the food pieces with a water-permeable polymeric material prior to the osmotic process [12,13,14,15]. The edible coating also improves the physicochemical and nutritional properties of dried fruits and vegetables by reducing oxygen diffusion into the food [16,17,18]. Edible films and coatings are prepared from a variety of polysaccharides, proteins, and lipid materials. Edible coatings should have good mechanical strength, satisfactory sensory properties, high water diffusivity, and low solute diffusivity [19]. Based on past studies, polysaccharides generally meet these requirements better than protein or lipid films and coatings alone [20]. Several studies have investigated the influence of coating types on the osmotic dehydration process and further drying performance [13,16,21,22,23]. A review on advances in edible coatings for fresh fruits and vegetables has been carried out by [19]; the reader is referred to this reference for an overview of edible coatings. The quest continues to develop a new-generation coating that incorporates active compounds using nanotechnological solutions, such as nanoencapsulation and multi-layered systems. Alginic acid and polygalacturonic acid are some of the suitable options for encapsulation. Alginic acid and polygalacturonic acid are the important derivatives for food applications. Furthermore, they are soluble in both hot or cold water and adaptable to a large scope of food elements, including proteins, sugars, and starches. The literature review revealed a clear shortcoming in the knowledge regarding using the proposed coating material. 

Substantial work has been done to establish models of atmospheric pressure to predict the osmotic dehydration process. However, there is a critical need to establish a mathematical model that takes into account all the factors associated in an osmotic drying (OD) process [24]. Empirical models are developed from experimental data. However, these models are only capable of representing data in a particular condition. Therefore, nonlinear models are more attractive for food processing due to the diversity and nonlinear behavior of natural products. 

Artificial neural network (ANN) models have recently gained a reputation in many fields. This method is highly efficient in solving the complex and nonlinear equations in dryers [25,26]. Various authors have recommended using ANN models for mass transfer kinetics during the OD process [27,28,29], because of the convolution of the osmotic dehydration process. Indeed, the literature review has highlighted an apparent lack of information regarding the application of ANN to model the OD process. Furthermore, no literature has been found on the effect of OD conditions on water loss, solid gain, and drying performance of potato and apple samples when using the ANN model for glucose and sucrose agents coupled with edible coatings. Therefore, a combined approach involving the OD process coupled with edible coatings and mathematical modeling is demonstrated in this work. More specifically, the specific objectives of this work are to determine the effects of coatings, namely alginic acid and polygalacturonic acid, on mass transfer during the osmotic dehydration process, and their effects on drying rate during convective drying. Additionally, an artificial neural network (ANN) is used to model the OD process and predict the drying performance. 

## 2. Material and Methods

### 2.1. Experimental Procedures

#### 2.1.1. Sample Preparation

Fresh potatoes and apples were bought from the supermarket and used as experimental materials in this study. Samples of the model materials were cut manually by an ultra-sharp knife into specific sizes. For potato, the size was 4 cm × 4 cm × 1.3 cm, with an initial weight of 25 g, while for apple, the size was 3.8 cm × 3.5 cm × 1.5 cm, with an initial weight of 15 g. The initial weights of the samples were taken before the samples were placed into the oven. The weights of the samples were measured using an electronic balance (accuracy ±0.01 gm). The samples were dried at 105 °C for 24 h and the bone-dry weights were measured upon completion of drying.

#### 2.1.2. Osmotic Solutions

Glucose and sucrose were dissolved in distilled water for use as osmotic agents. The solute contents varied from 30% to 50% (*w*/*v*) for both agents. Fully concentrated solutions were also used for investigation. For example, a 30% glucose solution was prepared by adding 75 g of glucose into 250 mL of distilled water. Glucose and sucrose, at concentrations such as 50% and full concentration, were also prepared following the same procedure. Alginic acid and polygalacturonic acid solutions with concentrations of 3% (*w*/*v*) were prepared using the same principle, mixed with 100 mL of distilled water [30].

#### 2.1.3. Osmotic Dehydration

The osmotic dehydration process with and without coating (alginic acid and polygalacturonic acid) was carried out for 2 h in both cases. Alginic acid (C14H22O13) is made from seaweed, which is composed of sodium alginate, calcium sulfate, and other ingredients that act as retarders in processed foods. Polygalacturonic acid, which is composed of galacturonic acid, in which the main component is pectin (65%), is used as a food additive.

The experiments were carried out by considering three main parameters, namely concentration, temperature level, and osmotic time. A beaker container made of glass was used to perform the OD process at a solution temperature of about 27 °C, at concentrations of 30% and 50% *w*/*v* for both glucose and sucrose solutions. Furthermore, a fully concentrated glucose and sucrose solution was also investigated. In each experiment, 6 samples were prepared. One sample was for initial bone dry mass, four were for the osmotic dehydration process, and one was for osmotic dehydration with drying. In the case of the experiment without coating, four samples were immersed in osmotic solutions. At every 30 min interval, one sample was taken out and the surface moisture was removed using a paper towel. The weights of the samples were then measured to determine the loss of moisture due to the osmosis process. The samples were then placed in the oven for 24 h to determine the bone dry mass. For the experiments with coatings, the samples were first dipped in the coating solution for 30 s then removed from the solution and dried for 5 min. Excess moisture on the surface of the samples was removed using a paper towel. Finally, the samples were immersed in the osmotic solutions for the osmotic dehydration process. The number of measured data is referred to as “n” in the following calculation, where it is necessary for standard deviation calculation. The experimental uncertainty for water loss and solids was within ±0.045%. The replicate experiment was conducted on a single sample and the data was within ±4%.

### 2.2. Air Drying

After the osmotic dehydration process, the convective drying experiment was carried out in an in-house built hot air drying system. The drying system was constructed measuring 60 cm length × 20 cm width × 20 cm height, using stainless steel for the drying chamber and an aluminum sheet as a diffuser. A tray with dimensions of 20 cm × 15 cm × 15 cm was made using an aluminum plate to place the samples on. A Philips Salon Dry Pro HP4991 hairdryer was used to produce a stream of hot, dry air, which was blown over the sample. The weight of the product was measured with an analytical balance (Model B-320C, Explorer OHAUS, Santa Clara, CA, USA) to an accuracy of ±0.0001 g. T-type copper–constantan thermocouples (Omega, Parsippany, NJ) were used to measure the preset drying temperature, with a noticeable accuracy level at about ±1.0 C. Temperatures were recorded using a data logger (Hewlett Packard 34970A, Lumberton, NJ, USA). For the bone dry mass of the product, an electric oven was used to dry the samples.

Prior to starting the drying process, the temperature of the drying system was set to 60 °C. For the drying process, the sample from the osmotic dehydration process was weighed and put on the tray. The tray was placed in the middle of the drying chamber. The drying process was carried out for 3 h. Samples were taken out every 15 min to measure the weight loss of moisture due to the drying process at that time. The time required to measure the weights of samples at different stages of drying was less than 30 s. The effect of the short interruption in comparison to the total drying time was negligible. The experimental uncertainty for moisture content was within ±0.029%. The reproducibility of the experiment was carried out under particular drying conditions, and the results were within ±5%.

### 2.3. C. Data Analysis

For osmotic dehydration, water loss (WL) and solute gain (SG) were calculated as follows:

WL (% wt) = ((M_o_ − M_d_) − (M_t_ − M_td_))/M_d_ (1)

SG (% wt) = (M_td_ − M_d_)/M_d_ (2)

where M_o_ is the initial mass of the material (g), M_t_ is the mass of the sample at time *t* (g), M_d_ is the initial bone dry mass of the sample material (g), and M_td_ is the bone dry mass of the sample at time *t* (g) during the osmotic dehydration process. 

For the drying experiment, drying rate (kg/m^2^ h), moisture content (g/g initial dry mass), and the dimensionless moisture content were considered and determined as follows:

Drying rate = (M_n_ − M_p_)/(0.25 × 1000A) (3)

MC = (M_n_ − M_fd_)/M_d_ (4)

DMC = MC_t_/MC_o_ (5)

where A is the surface area of the sample (m^2^), M_n_ is the mass of the sample at *t* time (g), M_p_ is the mass of the sample at the previous time *t* (g), M_fd_ is the final bone dry mass of the sample upon osmotic dehydration (g), M_d_ is the initial bone dry mass of the sample (g), MC_t_ is the moisture content of the sample at time *t* (g), and MC_o_ is the initial moisture content of the sample (g) before drying.

## 3. Artificial Neural Network (ANN)

**Figure 1:**
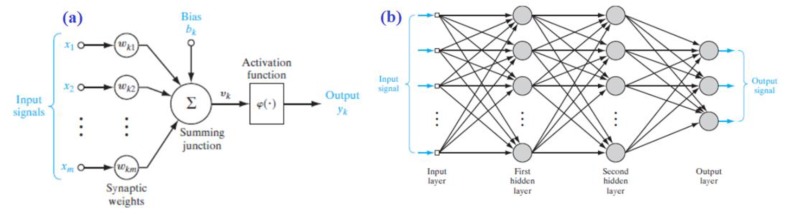
(**a**) Nonlinear model of a neuron (labeled k). (**b**) Architectural graph of a multilayer perceptron with two hidden layers.

Operations in the brain are executed through very tiny neural cells called neurons. The human brain contains a very large number of these cells, numbering approximately 100 billion cells [31]. Every neuron acts as a basic processing element in brain operations. These cells are connected through their synapses and dendrites to form a network. The neuron activates an electrochemical synaptic output as a function of the accumulated weighted input signals to its soma, which are received through the cell’s dendrites. However, the activation function can take the linear or nonlinear form. The output signal is then propagated to the other interconnected cells via the cell’s axiom and synapses. The model for learning ANN is shown in Figure 1a. Therefore, the perceptron output can be formed mathematically, as in the following equations:

(6) $$u_{k}=\sum_{j=1}^{m}\omega_{k_{j}}x_{j}$$

(7) $$y_{k}=\Phi \left(u_{k}+b_{k}\right)$$

where *y_k_* is the output of the *k*th neuron, *u_k_* is the accumulated weighted input, *w_k_j__* is the *j*th synaptic weight of the *k*th neuron, *x_j_* is the *j*th input, *b_k_* is the constant bias of the *k*th neuron, and *Φ* is the activation function.

The perceptron model was then developed to form a multilayer perceptron, or what is well-known now as an artificial neural network (ANN), as illustrated in Figure 1b. There are many architectures for ANNs; however, the most popular one is the feed-forward type [32]. The first and last layers are the input and output layers, respectively. In between the two layers, the hidden layers are located. The number of neurons in each layer is a user-defined and problem-dependent process, while the number of inputs and outputs defines the number of neurons in the first and last layers, respectively. Every neuron in the hidden and output layers is connected to the outputs of the neurons of the preceding layer. The synaptic weight of every connection defines the connection strength between the two neurons. The values for these weights have to be adjusted properly to satisfy the required model. The weights are adjusted through a learning algorithm. The most popular supervised learning algorithm for the ANN is backpropagation. 

## 4. Results and Discussions

### 4.1. Choice of Better Osmotic Solutions

Mass transfer during osmotic dehydration of potato samples was performed experimentally using five different concentrations of osmotic solutions, as presented in Table 1.

As reported in Table 1, a remarkable increase in WL was obtained using 50% concentrated and fully concentrated solutions compared to 30% concentrated solutions. However, there was only a slight difference in WL between 50% concentrated and fully concentrated solutions. It is likely that as the concentration of the solution gets higher, it creates a higher pressure gradient that acts as a driving force to increase the migration rate of water from the product to the solution. In the case of SG, it also shows that the solid gain increases with the increase of osmotic solution concentration. However, the increasing rate drops significantly at or higher than 50% concentrated solutions. This could be attributed to the fact that the formation of a protective film on the surface of the material restricts the solute permeability into the material from the surroundings. Overall, 50% glucose solution resulted in the best combination of WL and SG (i.e., produced higher WL and lowered SG). Therefore, this 50% glucose solution was considered to be the optimal osmotic condition, and thus we further continued with coating experiments using this osmotic condition. Several other studies also found that the osmotic dehydration of fruit was influenced by various process parameters, including osmotic matter and the concentration of an osmotic solution [12].

### 4.2. Effect of Coating on WL, SG and Drying Rate

**Figure 2:**
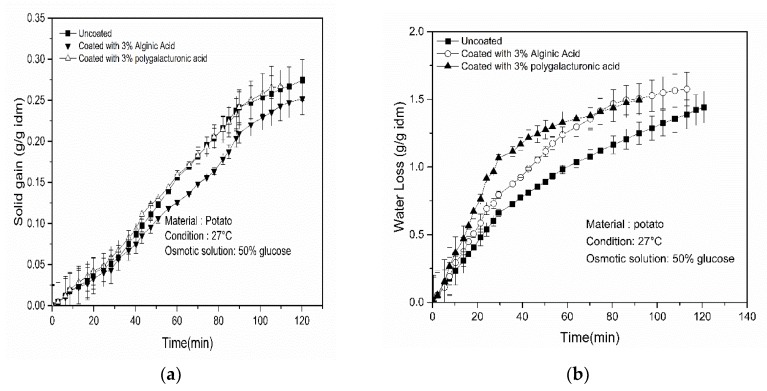
Variation of (**a**) water loss and (**b**) solid gain with time for potato sample.

The effect of a coating of alginic acid and polygalacturonic acid on potato in terms of water loss is shown in Figure 2a. Results revealed that samples with both coatings show higher WL compared to uncoated samples during the osmotic dehydration process. Final water loss after 120 min of the osmotic process was found to be 1.571 g/g idm (where idm is the initial dry mass) and 1.492 g/g idm for 3% alginic acid and 3% polygalacturonic acid, respectively. 

These results agreed with the results from other work [13], which stated that the coated sample slightly increased the rate of water removal. Figure 2b represents the solid gain for osmotically treated coated and uncoated potato and apple samples. The application of coating had an effect on the solid gain of the fruits/vegetables during osmotic dehydration [20]. It was observed that generally, the coated samples showed a lower solid gain compared to the uncoated samples, however, within the samples coated with two different coating materials, there was no significant difference in SG. Practically, the solid gains for the samples coated with 3% alginic acid and 3% polygalacturonic acid were 0.267 g/g idm and 0.269 g/g idm, respectively, which were almost the same. The lower SG of the coated samples could be due to the phenomenon that the coating behaved as an artificial barrier on the sample surface and thus effectively obstructed the penetration of solute into the samples. Another study [13] also observed similar findings.

**Figure 3:**
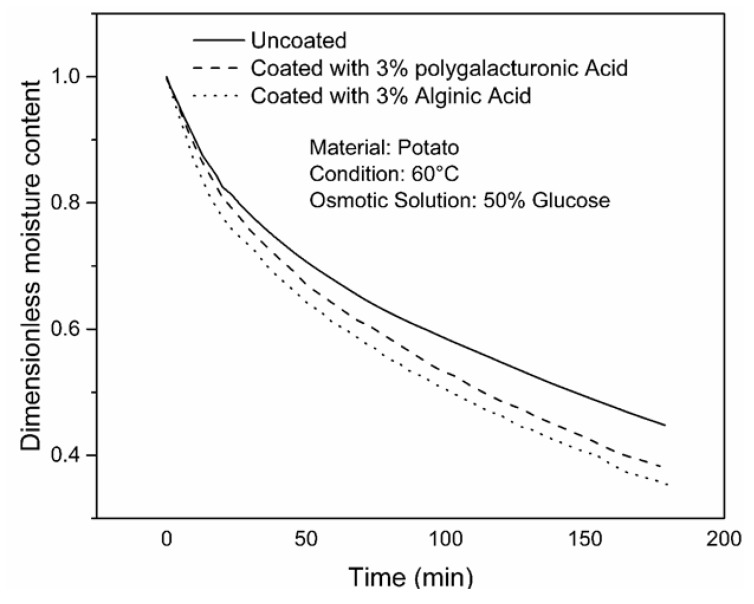
Variation of dimensionless moisture content with drying time.

The effect of coating on drying is demonstrated in Figure 3 in the form of dimensionless moisture content for better comparison. The results showed lower moisture content of the coated samples compared to the uncoated ones. The ultimate dimensionless moisture contents of the samples for uncoated, coated with 3% alginic acid and coated with 3% polygalacturonic acid were 0.448, 0.352, and 0.378, respectively, from the same initial dimensionless moisture content of 1 after a drying period of 3 h. As stated earlier, coated samples resulted in a lower solid gain compared to the uncoated samples. It is likely that the coating material hindered the development of concentrated solid layer under the surface of the product, which in return increased the driving force for water flow from inside the product to the surface of the product. 

**Figure 4:**
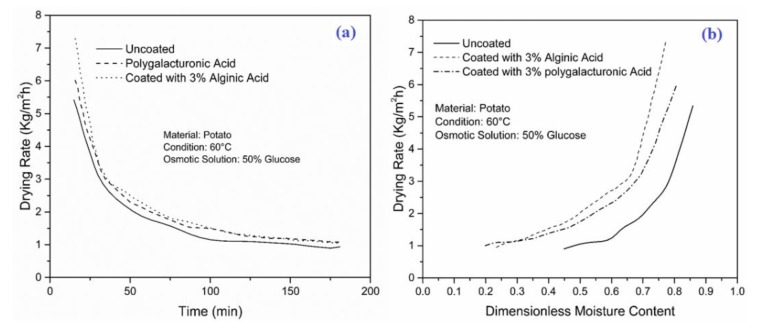
Variations of (**a**) drying rate with dimensionless moisture content and (**b**) time for potato samples.

The variation of drying rate with time for osmotically treated potato samples coated with both coating materials and uncoated samples is presented in Figure 4a. The results revealed that the samples with both coated materials showed a higher drying rate compared to the uncoated samples. Similarly, Figure 4b shows the variation of drying rate with dimensionless moisture content between uncoated and coated samples. A noticeable improvement was observed in the drying rate for the samples coated with both coating materials compared to the uncoated samples. Results also revealed that the sample coated with 3% alginic acid showed a higher drying rate than the samples coated with 3% polygalacturonic acid. This can be attributed to the fact that coated samples reduced the solid gain, and in turn increased the driving force for water flow from inside the product to the surface of the product. Therefore, this increased the migration of moisture from inside to the surface of the product, and hence increased the drying rate. 

**Figure 5:**
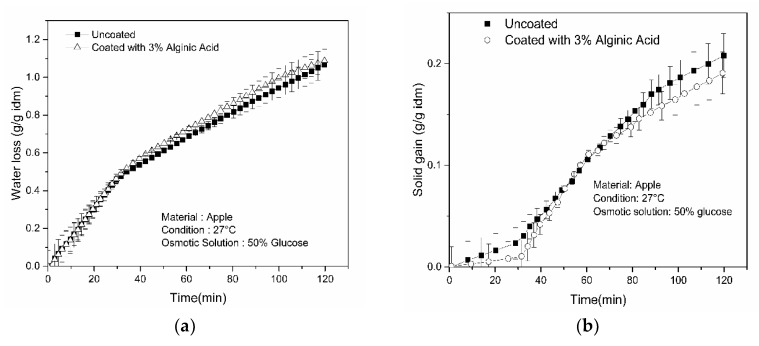
Variations of (**a**) water loss and (**b**) solid gain with time for apple samples.

Figure 5a shows the variation of water loss between the uncoated apple samples and those coated with 3% alginic acid. Higher WL was obtained for the coated samples compared to the uncoated samples. Upon 2 h of drying, the ultimate WL was measured to be 1.069 g/g idm and 1.124 g/g idm for the uncoated and coated samples, respectively. These results showed an improvement of about 5% WL during a 2 h osmotic dehydration process.

**Figure 6:**
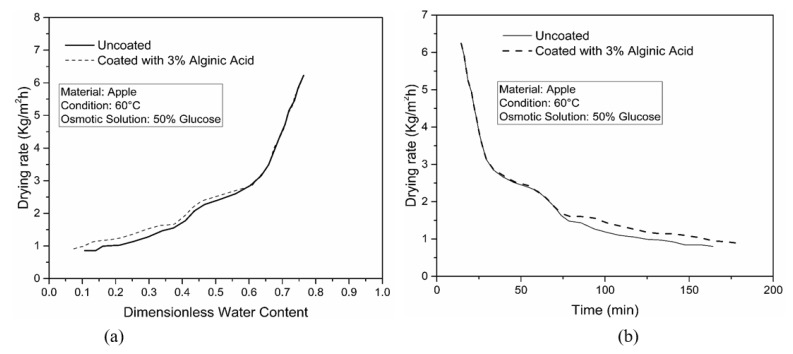
Variations of (**a**) drying rate with dimensionless moisture content and (**b**) time for apple samples.

Furthermore, a significant improvement in final solid gain was also observed, as demonstrated in Figure 5b, for osmotically treated coated apple samples. The final solid gain for apple sample coated with 3% alginic acid was 0.192 g/g idm compared to the uncoated sample, which was about 0.208 g/g idm after 2 h of the osmotic dehydration process. This can be attributed to the fact that the coating acted an artificial barrier on the surface, and thus effectively obstructed the penetration of solute inside the sample. The investigation was also conducted by using apple samples, as shown in Figure 6. As with potato samples, similar patterns of results were observed (i.e., sample coated with 3% alginic acid showed enhanced drying performance compared to uncoated samples).

### 4.3. C. ANN Based Model

**Figure 7:**
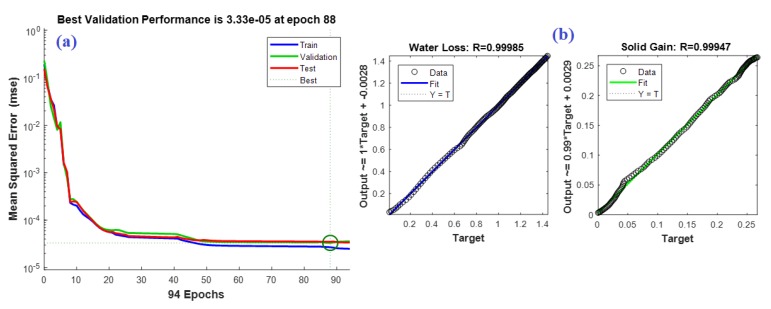
(**a**) Training performance of the potato artificial neural network (ANN) model. (**b**) Prediction accuracy of the potato ANN model.

**Figure 8:**
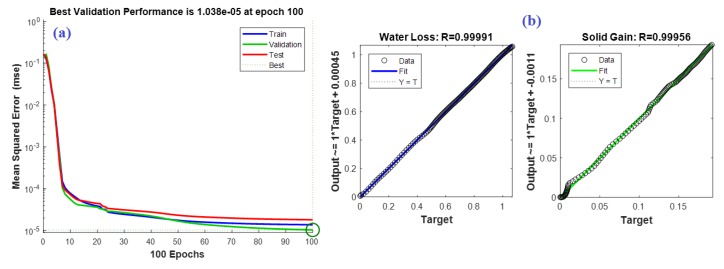
(**a**) Training performance of the apple ANN model. (**b**) Prediction accuracy of the apple ANN model.

The ANN has proved its efficiency in many engineering applications, especially in modeling problems [33]. Therefore, the data collected from the fruit drying experiments with and without acidic coating were used to build an ANN model for both the potato and apple. The input variables of the potato model were the time (minutes), the alginic acid (%), and the polygalacturonic acid (%), and the outputs were the water loss (g/g idm) and the solid gain (g/g idm). However, the same potato model’s inputs, except for the polygalacturonic acid (%), were the inputs for the apple model, and the outputs were the same as the potato model. Therefore, (3 4 4 2) and (2 4 4 2) are the feed-forward ANN structures of the potato and apple models, respectively. Here, (3 4 4 2) implies that the ANN has 3 inputs, 2 hidden layers, and 2 outputs. The two hidden layers for both models each contain only four neurons. The hidden and output layers’ activation functions are “tansig” and “linear” types, respectively. The training-to-testing ratio was 60:40 and the models were trained using the back-propagation learning algorithm for 100 epochs. The models were trained until a minimum error for the testing data was reached. The mean squared errors (MSEs) and the R^2^ were used as statistical measures to assess the modeling performance. The obtained MSEs for the predicted water loss and solid gain outputs of the potato model were 4.0948e^−5^ and 3.924e^−6^, respectively. However, they were 3.164e^−5^ and 4.4915e^−6^ for the apple model. The *r*^2^ values for the two outputs of the potato model were found to be 0.99969 and 0.99895, respectively, while they were 0.99982 and 0.99913 for the apple model, which reinforces the modeling phase. Figure 7 and Figure 8 show both models’ training performances and prediction accuracies.

**Figure 9:**
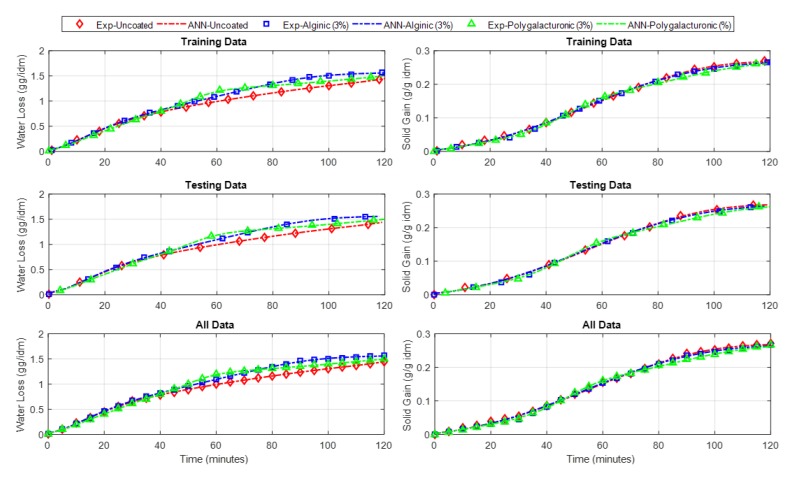
Experimental and ANN outputs of the water loss and solid gain of the potato model.

**Figure 10:**
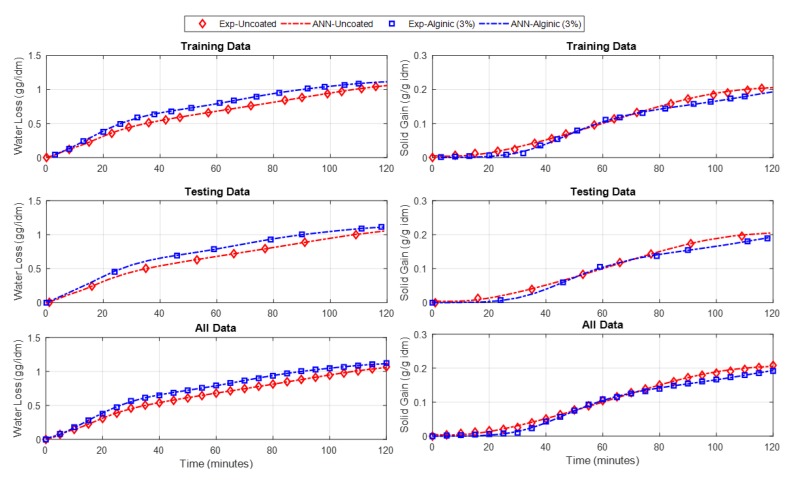
Experimental and ANN outputs of the water loss and solid gain of the apple model.

The resulting outputs are plotted for the ANN models compared to the experimental data of the potato and apple models, as shown in Figure 9 and Figure 10, respectively. 

**Figure 11:**
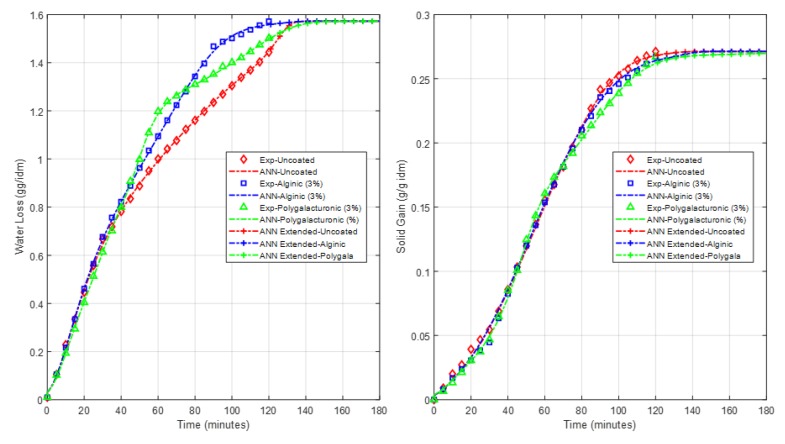
Potato model predictions, including the extended time.

**Figure 12:**
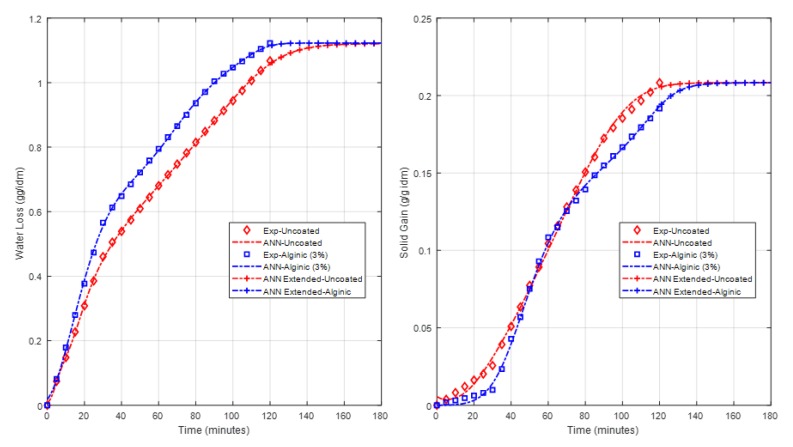
Apple model predictions, including the extended time.

The predictions of the two models were extended to obtain the outputs of both models beyond the 120 min of experimental data. The predictions were extended for 60 min after the experimental data time for all uncoated and coated cases. Figure 11 and Figure 12 illustrate the plots of the model predictions, including the response of future extension of the potato and apple models, respectively. The models’ extended predictions can definitely give a picture of how the response is going after the time of the collected experimental data.

## 5. Conclusions

A study on the influences of two suitable coating materials (alginic acid and polygalacturonic acid) on the osmotic dehydration and drying performance of convective air drying of potatoes and apples was successfully conducted. Results demonstrated that both coating materials enhanced osmotic dehydration, causing higher water loss and lower solid gain of the vegetable and fruit products. A remarkable enhancement of drying performance was also observed. Furthermore, a comparative study between the two coating materials demonstrated that alginic acid showed better performance compared to polygalacturonic acid. Finally, results revealed that the proposed new coating materials (alginic acid and polygalacturonic acid) hold high potential for the pretreatment method prior to the drying process for fruit and vegetable products. Moreover, a robust model of the drying process was created based on the experimental dataset, employing an artificial neural network. The obtained Mean Square Errors (MSEs) for the predicted water loss and solid gain outputs of the potato model were 4.0948e^−5^ and 3.924e^−6^, respectively. However, these values were 3.164e^−5^ and 4.4915e^−6^ for the same parameters in the apple model. The R^2^ values for the two outputs of the potato model were 0.99969 and 0.99895, respectively, while they were 0.99982 and 0.99913 for the apple model, which reinforces the modeling phase. Therefore, the developed ANN model was suitable for predicting water loss and solid gain during the OD process, as well as enhanced the drying performance of convective hot air drying.

## Figures and Tables

**Table 1:** Water loss (WL) and solids gain (SG) in different osmotic solutions.

Solution	Water Loss (g/g idm)	Standard Deviation	Solid Gain (g/g idm)	Standard Deviation
30% Glucose	1.056	0.07	0.219	0.02
50% Glucose	1.442		0.275	
30% Sucrose	1.011	0.05	0.209	0.03
50% Sucrose	1.438		0.283	

* Idm: initial dry mass.
